# A systematic analysis of the RNA-targeting potential of secreted bacterial effector proteins

**DOI:** 10.1038/s41598-017-09527-0

**Published:** 2017-08-24

**Authors:** Caroline Tawk, Malvika Sharan, Ana Eulalio, Jörg Vogel

**Affiliations:** 1 Institute for Molecular Infection Biology, University of Würzburg, Würzburg, Germany; 2 Helmholtz Institute for RNA-based Infection Research (HIRI), Würzburg, Germany; 3 Present Address: European Molecular Biology Laboratory (EMBL), Heidelberg, Germany

## Abstract

Many pathogenic bacteria utilize specialized secretion systems to deliver proteins called effectors into eukaryotic cells for manipulation of host pathways. The vast majority of known effector targets are host proteins, whereas a potential targeting of host nucleic acids remains little explored. There is only one family of effectors known to target DNA directly, and effectors binding host RNA are unknown. Here, we take a two-pronged approach to search for RNA-binding effectors, combining biocomputational prediction of RNA-binding domains (RBDs) in a newly assembled comprehensive dataset of bacterial secreted proteins, and experimental screening for RNA binding in mammalian cells. Only a small subset of effectors were predicted to carry an RBD, indicating that if RNA targeting was common, it would likely involve new types of RBDs. Our experimental evaluation of effectors with predicted RBDs further argues for a general paucity of RNA binding activities amongst bacterial effectors. We obtained evidence that PipB2 and Lpg2844, effector proteins of *Salmonella* and *Legionella* species, respectively, may harbor novel biochemical activities. Our study presenting the first systematic evaluation of the RNA-targeting potential of bacterial effectors offers a basis for discussion of whether or not host RNA is a prominent target of secreted bacterial proteins.

## Introduction

Many bacterial pathogens depend on virulence factors, translocated into the host by specialized secretion systems, to subvert distinct cellular functions. The number of these secreted proteins, known as effectors, encoded by a pathogen varies greatly from one to several hundred depending on the species^1^. The characterization of such effectors promises both, a better understanding of the infection processes and new applications in cell biology and biotechnology. To date, almost all of the identified targets of effectors are host proteins^2, 3^, prominently involved in infection-related processes such as cytoskeletal manipulation, immune-evasion, apoptosis signaling, and vesicle trafficking^4, 5^. Effector proteins from widely divergent pathogens may target similar proteins and pathways in the host^3, 6^, with the NFκB innate immune pathway^7^ being a prominent example. Similarly, small GTPases are commonly targeted to reroute vesicular trafficking avoiding lysosomal degradation and favoring intracellular replication of bacteria^8^. Understanding the functions of effector proteins is essential to unravel the mechanisms of bacterial survival in the host.

By contrast, targeting of host nucleic acids by effectors remains little explored. One example are transcription activator-like effectors (TALEs) of *Xanthomonas* (a plant pathogen) in which nearly-identical repeats containing a hypervariable pair of residues confer specificity to a particular base pair, allowing them to bind to promoters in host DNA^9–11^. In addition to TALEs, effectors harboring SET-like domains influence host gene expression by modifying chromatin state^12–14^.

Beyond DNA, cellular RNA would offer a wide spectrum of potential targets for effectors to interfere with host gene expression post-transcriptionally at the levels of RNA splicing, maturation, export, silencing, and storage. Endogenous RNA-binding proteins (RBPs) play an essential role in these processes and there is an increasing number of new proteins with RNA-related function^15^. Therefore, it was conceivable that some bacterial effectors possess RNA-binding domains (RBDs), which would allow them to selectively target coding and non-coding RNAs to modulate host gene expression, similarly to viral encoded RNA-binding proteins^16–18^. Additionally, viruses are known to directly manipulate the host microRNA pathway by this mechanism^19^. However, in contrast with the importance of hijacking host RNA-mediated regulation by viral proteins, no RNA-targeting bacterial effectors have been identified so far. The only identified RNA-binding effector to date is the *Yersinia* effector YopD, which regulates T3SS genes by binding directly to bacterial mRNAs at short AU-rich sequences^20, 21^.

Many characterized effectors target specific host pathways by mimicking host proteins, hence very often they contain eukaryotic-like domains^2^. This suggests that RNA-targeting effectors may be identified by homology searches for conserved RBD domains. Indeed, automated homology-based approaches have enabled RBP discovery in various organisms^15, 22^. With the recent characterization of hundreds of RBPs, a large collection of RBDs is now available. For instance, the Pfam database now contains ~800 RBDs including RNA-binding protein families^23^. This rich collection of domains lends itself as a reference set for the prediction of potential RNA-binding effectors.

The recent increase in known RBPs was much driven by novel methods for the exploration of RNA-protein interactions^24, 25^. Collectively, these studies have identified ~1,500 RBPs in human cells, which contain ~600 distinct RBDs, many of them widely conserved whereas the rest are novel domains with unique architecture^15^. Methods using protein or RNA as bait combined with high-throughput RNA sequencing and mass spectrometry have accelerated RBP discovery^25, 26^. Moreover, *in vivo* UV crosslinking combined with immunoprecipitation (CLIP) not only permits the capture of endogenous RNA-protein interactions but also pinpoint interaction sites^24, 27–30^. This approach allows the purification of the cross-linked RNA-protein pairs under harsh washing conditions, which reduces false-positive interactions with non-specific RNA^31^.

In this study, we have assembled from the literature a searchable collection of 1,022 individual effector proteins of 35 animal and plant pathogens or symbionts. To identify RBPs in this effector dataset, we applied a biocomputational approach that used as a reference all currently identified unambiguous RBDs. This analysis predicted several classical and non-classical RBDs in a small subset of effector proteins. We applied the CLIP method to interrogate 33 selected effector proteins with putative RBDs for their RNA binding potential. Our combined biocomputational/experimental approach unraveled important limitations and challenges both in the automated prediction of RBDs in bacterial effector proteins using sequence-based homology, as well as in the CLIP approach when used with a broad diversity of proteins. For example, UV irradiation, use of ATP [Υ-^32^P] and co-purification of proteins, if not considered with adequate controls, can mislead in the interpretation of the results. Nonetheless, our results also suggest that the long-studied *Salmonella* effector PipB2 and the uncharacterized *Legionella* effector Lpg2844 may harbor a yet unidentified nucleotide-binding domain.

## Results

### A bioinformatics approach to predicting RBD-like domains in bacterial effectors

**Figure 1:**
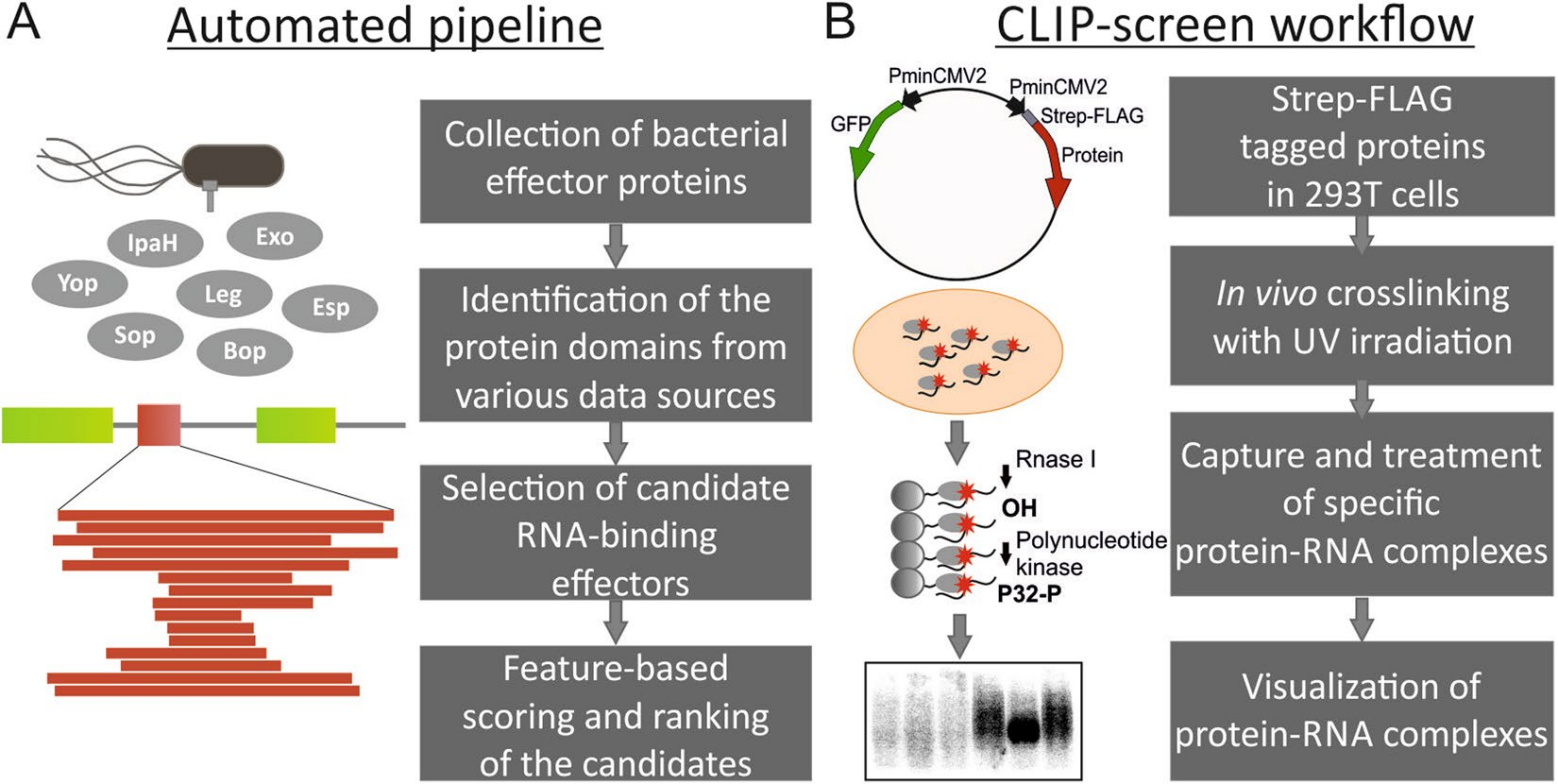
Schematic representation of the combined computational and CLIP-screening approach to identify RNA-binding effectors (**A)**. The main steps involving the computational analysis by APRICOT pipeline. A comprehensive list of all the available secreted effector proteins was assembled for the identification of RBDs. RNA-binding domains were identified using various domain databases and the associated domain search algorithms assembled. The predictions were ranked using feature-based scoring (See materials and methods). (**B)** Selected candidates were cloned into a bidirectional plasmid with an N-terminal Strep-FLAG tag and expressed in HEK293T cells. Proteins were analyzed using the CLIP-screening approach. The main steps of the CLIP-screening workflow are indicated. Cells expressing the effectors were UV-crosslinked *in vivo*, the protein-RNA complexes purified and subjected to enzymatic treatments, then visualized after separation on a gel.

A combination of *in silico* automated prediction and a CLIP-screening approach was developed to capture RNA-protein interactions between bacterial effectors and host RNA (Fig. 1). For the prediction of RBDs in effectors, we manually assembled a dataset comprising all known and hypothetical effector proteins (gene names and amino acid sequences). Individual secreted effectors were collected from the literature, databases, and small-scale or global screening studies. Upon manual curation, we obtained a non-redundant dataset of 1,022 unique proteins corresponding to one representative genus for each of 35 animal and plant bacterial pathogens or symbionts; except for *Pseudomonas* for which two representative genera for each of the plant and animal pathogens were included because of their distinct repertoire of effectors (Supplementary Tables S1 and S2). Of note, the dataset includes proteins predicted as secreted effectors by machine-learning approaches or found by reporter screens, some of which might represent false-positives to be eliminated upon new evidence (Supplementary Table S2). In addition to the gene names and protein sequences of effectors, available data on function, localization, homology, and other features were assembled resulting in an ample resource summarizing findings on bacterial effector proteins and their functions (Supplementary Table S2).

In order to identify among the compiled list of effectors, the ones bearing putative RBDs we ran them through an in-house developed bioinformatics pipeline called APRICOT^32^ (Fig. 1A). The first step of this analysis was to assemble a reference dataset of RBDs from available RNA-protein interactome studies. This culminated in 44 classical and 68 non-classical RBDs, where classical RBDs are domains found in RBPs commonly involved in RNA metabolism, and the non-classical domains are proven to bind RNA but do not have a putative RNA-related function (Supplementary Table S3). The collection was limited to 112 RBDs to avoid ambiguous domains that would potentially give a large number of false-positives. The 112 domains were extracted by stringent keyword searches from two main data sources Conserved Domain Database (CDD) and InterPro. This was followed by the prediction of global domain conservation in the effectors using the domain models Hidden Markov Model (HMM) and Position-Specific Scoring Matrix (PSSM) available in the CDD and InterPro databases. The predictions were scored using multiple parameters, including domain coverage, percentage similarity, E-value, and percentage identity and finally ranked according to these feature-based scores. The optimal cutoff values were determined using large training datasets of positive and negative RNA-binding proteins^32^, and the ranked predictions were then manually curated to obtain a final set (Supplementary Table S5).

The majority of the functionally characterized domains of effectors bear some similarity to their eukaryotic counterparts, which can vary from the entire domain sequence or domain-architecture to the conservation of only catalytic residues (Tables 1 and 2). To assess the extent of conservation with eukaryotic domains, we compiled a list of 12 previously studied effectors from *E.coli*, *Salmonella*, *Shigella*, *Legionella*, and *Yersinia* with characterized domains mimicking a host protein. The corresponding eukaryotic domains of 9 of the 12 selected proteins were identified using the APRICOT computational pipeline^32^ (Table 1, Supplementary Table S4). For YopJ, IpgD, and SopA no homology to eukaryotic domains was identified, and for DrrA and SopB only distantly related domains were identified (Table 1). This may be explained by a previously observed low sequence conservation of these effectors; SopB and its orthologue IpgD both possess phosphatidylinositol phosphatase activity, but are related to the eukaryotic counterpart by only a few residues in the active site, particularly a catalytic cysteine^33^. Similarly, DrrA functions as a guanine nucleotide exchange factor (GEF) but there is no identifiable homology with any known GEFs^34^. SopA and YopJ do not have identifiable sequence similarities to HECT E3 ligases or cysteine proteases respectively, except for a crucial catalytic cysteine residue^35, 36^. These examples emphasize a limitation in identifying domains having very little similarity with the eukaryotic domain. Interestingly, the remaining 7 positive domains showed consistently high coverage and similarity values (>40%), and low E-values (<0.5) (Table 1, Supplementary Table S4). The analysis of this test set demonstrates that effectors with a domain similarity to eukaryotic proteins that exceeds a few catalytic residues, can be identified using our automated pipeline and parameters. In addition, it revealed some limitations related to low conservation (e.g. SopB, DrrA, YopJ, IpgD, and SopA; Table 1).

**Table 1:** Prediction of characterized eukaryotic domains

Organism	DomainID	FullName	Evalue	% Coverage	% Similarity
*Escherichia*	NleH1	Kinase domain, and C-terminal PDZ-binding motif^69, 70^			
	SSF56112	Protein kinase	1,5E-05	—	—
*Salmonella*	**steC**	**Eukaryotic-like kinase** ^71^			
	cd00180	Catalytic domain of Protein Kinases	0,07	54,4	22,3
	PF00069	Protein kinase domain	0,04	45,4	21,5
*Salmonella*	**sopB**	**Phosphoinositide phosphatase (PiPase)** ^72^			
	cd09562	SAM domain of liprin-alpha1,2,3,4 proteins repeat 1. Liprins were originally identified as LAR (leukocyte common antigen-related) transmembrane protein-tyrosine phosphatase-interacting proteins.	1,5	45,1	29,6
	PF13350	Tyrosine phosphatase family	2,5	15,2	9,1
*Yersinia*	**yopH**	**Protein tyrosine phosphatase (PTPase)** ^73^			
	PF00102	Protein-tyrosine phosphatase.	7E-39	100,0	43,5
	cd00047	Protein tyrosine phosphatases (PTP)	1E-45	101,3	43,7
*Salmonella*	**spvB**	**ADP-ribosyltransferases** ^74^			
	PF03496	This entry represents an ADP-ribosyltransferase domain found in various proteins	2,9E-14	82,3	—
*Yersinia*	**yopJ**	**cysteine protease, ubiquitin-like protein protease** ^75^			
		None found			
*Shigella*	**ipgD**	**Phosphoinositide phosphatase (PiPase)** ^76^			
		None found			
*Legionella*	**drrA**	**guanine nucleotide exchange factors (GEFs)** ^34^			
	cd07660	BAR domain of Arfaptin. Arfaptins are ubiquitously expressed proteins implicated in mediating cross-talk between Rac, a member of the Rho family GTPases, and Arf (ADP-ribosylation factor) small GTPases.	4,8	41,8	19,9
*Legionella*	**setA**	**N-terminal glucosyltransferase domain and a C-terminal phosphatidylinositol 3-phosphate-binding domain** ^77^			
	PF04488	Glycosyltransferase sugar-binding region containing DXD motif	6E-06	100,0	34,4
*Legionella*	**ralF**	**guanine nucleotide exchange factors (GEFs)** ^78^			
	PLN03076	ARF guanine nucleotide exchange factor (ARF-GEF)	0	10,3	6,3
	cd00171	The Sec. 7 domain is the central domain of the guanine-nucleotide-exchange factors (GEFs) of the ADP-ribosylation factor family of small GTPases (ARFs)	8E-60	100,5	63,2
*Shigella*	**ipaH9.8**	**Variable N-terminal region containing LRRs and a highly conserved C-terminal region that contains the novel E3 ligase domain** ^79^			
	COG4886	Leucine-rich repeat (LRR) protein [Transcription].	4E-18	76,4	25,1
	sd00033	leucine-rich repeats, ribonuclease inhibitor (RI)-like subfamily	2E-13	71,0	40,3
*Salmonella*	**sopA**	**Homology to eukaryotic HECT E3 ligases** ^36^			
		None found			

Twelve effector proteins with characterized eukaryotic-like domains were selected and submitted for domain prediction with APRICOT. The organisms, effector protein names, the characterized domains, and the reference studies are highlighted in grey and bold. The results and scores from the automated prediction are below each effector in white. ‘−’Indicates value not available.

Our APRICOT-based analysis of 1,022 effectors most frequently (13 out of 44) predicted the classical RBDs RRM, KH, DEAD, La, PUA, HA2, RGG, tRNA synt, KOW, S4, RAP, RNase and S1. Non-classical domains were predicted, too: ribosomal, SAM, WD40, THUMP, R3H and GTP. RBDs were predicted in 147 proteins out of 1,022 effectors analyzed. Among these, 88 proteins had prediction scores above the defined threshold for relevant RNA-related domains (coverage 25–35%, similarity 20–25%, E-value < 0.5; Supplementary Table S5). These candidates were then manually curated to exclude false-positives. For example, a cyclophilin-RRM and a WD40 domain were predicted for the *Legionella* effector Lpg1962, but the former domain was excluded. That is, when aligned with a representative consensus sequence of a cyclophilin containing an RRM, the Lpg1962 sequence did not overlap with the RRM region but rather with the protein-protein interaction domain of the cyclophilin (Supplementary Figure S1). Nonetheless, this protein was selected for further analysis due to the presence of the WD40 domain, recently established as a non-classical RBD^28, 37^.

Structural information helped to further exclude false-positives. For example, the *Burkholderia* BipD protein showed high-coverage (83%) and homology to the classical KH RNA-binding domain of Polynucleotide Phosphorylase (PNPase). Comparison of the BipD structure (PDB 2J9T^38^;) with a reference PNPase structure (PDB 3GCM^39^;), clearly showed that the BipD does not overlap with the RNA-binding domain of PNPase (Supplementary Figure S1). Importantly, the structural information revealed promising candidates. Comparison of the available structures of the effector E3 ubiquitin ligases YopM, IpaHs, and SspHs (Protein Data Bank^40^), predicted some overlap with the tertiary structure of TLR receptors, some of which are nucleic acid-binding (e.g. TLR3, TLR7, TLR8, and TLR9). Superimposing the structures revealed a possible overlap of SspH2, YopM, and IpaH3 with the ligand-interacting domain of the human and mouse Toll-like receptor 3 (Supplementary Figure S2). The alignment occurred at the level of the leucine-rich repeats regions, revealing a clear conservation of arginine and lysine residues; such residues are known to be important for RNA binding (Supplementary Figure 2; ref. 41). Notably, YopM, SspH1, and IpaH9.8 are targeted to the nucleus where multiple RNA-regulatory processes occur^42–44^. IpaH9.8 was particularly interesting, given that an RRM was predicted for this protein (Table 2; Supplementary Table S5).

**Table 2:** Selected effector candidates

Protein	Predicted domain name	Domain category	Domain type	E-value	Similarity %	Coverage %	Species
legAS4 /Lpg1718	RNA recognition motif-like Smg4_UPF3	RRM	Classical	0.15	29.55	61.36	*Legionella*
legC2	Ribonuclease Y	RNAse Y	Non-Classical	0.00	15.18	33.85	*Legionella*
legC8	Putative RNA-associated protein	RNA_bind	Non-Classical	1.40	12.55	27.71	*Legionella*
legL1	Leucine rich repeat, ribonuclease inhibitor type	RNAse	Non-classical	8.20	—	—	*Legionella*
lepA	Predicted RNA-binding protein	RNA_bind	Non-Classical	0.13	8.87	26.24	*Legionella*
lepB	Ribonuclease Y	RNAse Y	Non-Classical	0.32	16.15	37.94	*Legionella*
lpg0191	La RNA-binding domain of La-related protein 4	La	Classical	0.68	22.67	41.33	*Legionella*
lpg1290	RNA recognition motif in U2 small nuclear	LSM	Classical	0.47	22.86	46.67	*Legionella*
lpg1489	Superfamily II RNA helicase	DEAD	Classical	0.52	4.42	12.10	*Legionella*
lpg1751	RNA polymerase sigma factor	possible RBD	Non-Classical	7.10	20.25	45.57	*Legionella*
lpg1962	Cyclophilin_RRM: cyclophilin-type	RRM	Classical	0.00	48.19	101.20	*Legionella*
lpg1962	Cyclophilin_WD40: cyclophilin-type	WD40	Non-Classical	0.00	—	—	*Legionella*
lpg2327	RRP7 domain ribosomal RNA-processing protein 7	Ribosomal	Non-Classical	4.70	22.66	46.88	*Legionella*
lpg2844	Ebola nucleoprotein	possible RBD	Non-Classical	0.62	8.65	31.52	*Legionella*
lpg2847	Leucyl/phenylalanyl-tRNA protein	tRNA	Non-Classical	7.10	9.25	20.81	*Legionella*
lpg2936	RNA methyltransferase	RNA methyl- transferase	Classical	0.00	—	—	*Legionella*
lpg2936	RNA methyltransferase	RNA methyl- transferase	Classical	0.00	60.00	99.58	*Legionella*
lubX	Zinc finger (Znf) domains	ZnFC2HC, zf-CCHC	Non-Classical	0,00	—	—	*Legionella*
lubX	Zinc finger (Znf) domains	ZnFC2HC, zf-CCHC	Non-Classical	5.10	21.21	29.29	*Legionella*
pipB	mRNA capping enzyme	mRNA capping	Classical	2.80	1.34	3.47	*Salmonella*
pipB2	Sm protein G	LSM	Classical	1.70	15.71	41.43	*Salmonella*
sipB	Alanyl-tRNA synthetase	tRNA_synth	Non-Classical	2.00	3.63	8.01	*Salmonella*
slrP	Leucine Rich Repeat	possible RBD	Non-Classical	0.42	—	—	*Salmonella*
sseK3	tRNA pseudouridine synthase B	PseudoU_synth	Non-Classical	6.30	10.26	35.90	*Salmonella*
sspH2	Leucine rich repeat	possible RBD	Non-Classical	0.19	—	—	*Salmonella*
sspH1	Leucine Rich Repeat	possible RBD	Non-Classical	0.02	—	—	*Salmonella*
vrgS	The phosphoinositide binding Phox Homology	Non-RBD		0.51	16.54	62.99	*Salmonella*
ipaC	Seryl-tRNA synthetase	Ribosomal	Non-Classical	0.01	6.76	20.75	*Shigella*
ipaH9.8	RNA recognition motif 2 of RNA-binding protein	RRM	Classical	0.28	46.15	101.28	*Shigella*
ospG	Poly(A) polymerase	PAP_assoc	Non-Classical	0.86	5.85	12.20	*Shigella*
IpaH3	Leucine Rich Repeat	possible RBD	Non-Classical	0.12	—	—	*Shigella*
ORF169b	Ribosomal protein L25	Ribosomal	Non-Classical	2.80	14.77	32.39	*Shigella*
yopB	NOP domain	Nop	Non-Classical	0.70	27.66	60.64	*Yersinia*
yopD	NR_LBD_ER_like	Non-RBD	Non-RBD	2.30	9.95	15.38	*Yersinia*
yopE	SUD-M, Single-stranded poly(A) binding domain.	PAM2	Classical	5.20	10.26	35.90	*Yersinia*
yopM	Leucine rich repeat	possible RBD	Non-Classical	0.25	—	—	*Yersinia*

Thirty-three candidate RNA-binding effector proteins were selected for screening. The table comprises the protein name, the predicted RNA-binding domain, domain category and domain type according to the selected 112 known RBDs, the prediction scores, and the bacterial species. ‘—’ indicates value not available, the corresponding candidates were selected individually (see main text).

### Crosslinking and immunoprecipitation (CLIP) to evaluate RNA-targeting effector candidates

CLIP has been extensively used for the study of RNA-protein interactions in various organisms^28, 30, 45^. UV-crosslinking leads to the formation of an irreversible covalent bond between closely interacting nucleotides and peptides, thus allowing the elimination of non-specific RNA^31^. We established a CLIP-based screening method for the fast and efficient identification of the RNA-binding capacity of proteins. To optimize the protocol, we chose five human RNA-binding proteins, namely TIA1 cytotoxic granule-associated RNA binding protein-like 1 (TIAR), pumilio RNA binding family member 2 (PUM2), La ribonucleoprotein domain family member 7 (Larp7), enolase 1 (ENO1), and serine hydroxymethyltransferase 2 (SHMT2). TIAR and PUM2 are well characterized RBPs with a large number of cellular RNA targets (Supplementary Table S6; refs 46, 47). Larp7 has been shown to mainly bind the 7SK small nuclear RNA, as part of the 7SK nuclear particle (Supplementary Table S6; ref. 48). ENO1 and SHMT2, two metabolic enzymes identified as RBPs in a recent PAR-CLIP study were chosen as representatives of non-classical RNA-protein interactions (Supplementary Table S6; ref. 28).

**Figure 2:**
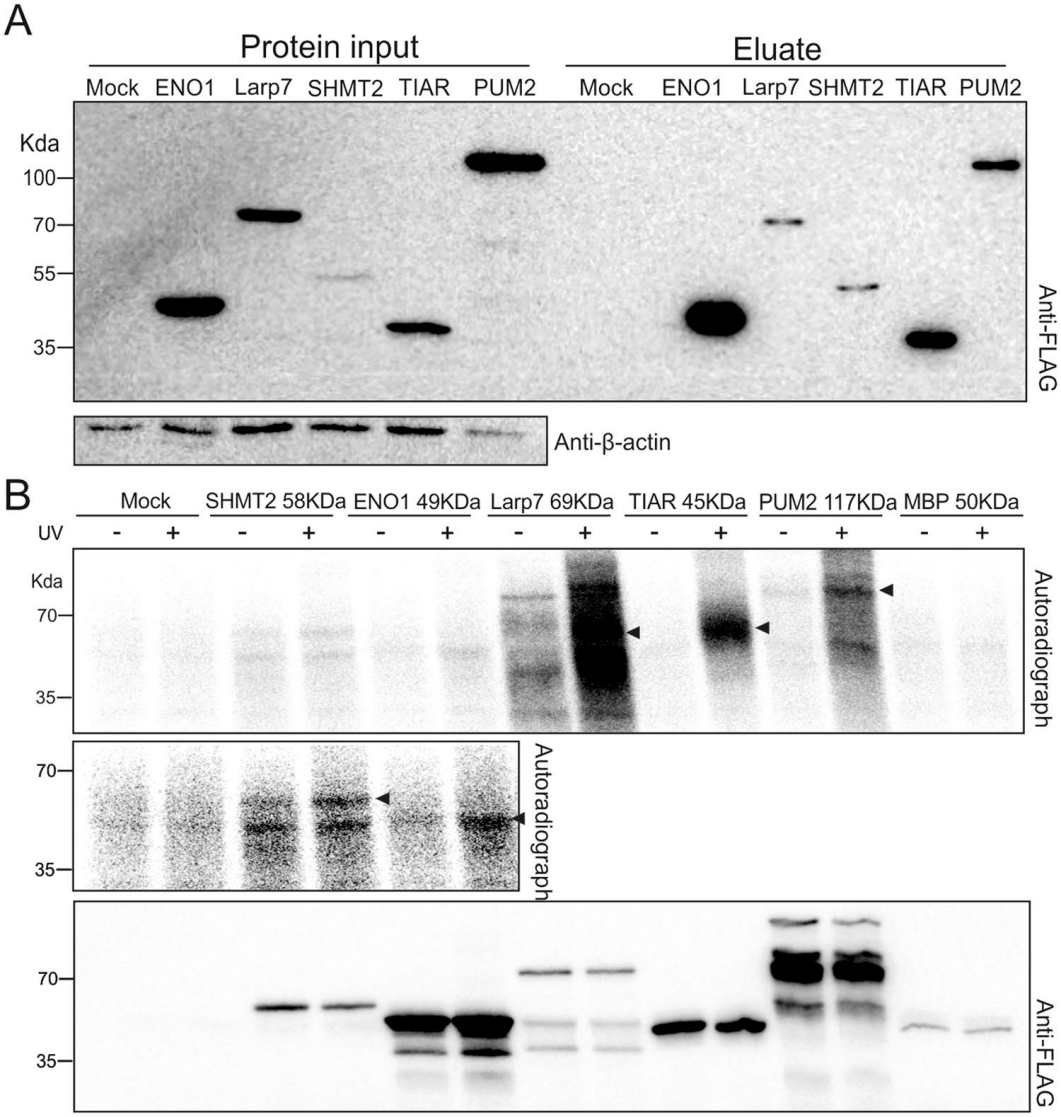
Development of the CLIP-screening workflow using an RBP test-set (**A)**. Detection on Western blot of Strep-FLAG tagged proteins, using an anti-FLAG antibody. Proteins are detected in the lysate (input) and after purification (eluate); all proteins are expressed and purified. The protein levels are compared to the endogenous β-actin levels. (**B)** Upper panel, autoradiograph showing the labelled RNA in association with the purified proteins. Arrowheads show the approximate molecular weight of the purified protein. Middle panel, an increased exposure of ENO-1 and SHMT2 signals. Lower panel, Western blot analysis detecting the tagged proteins in the purified complexes using an anti-FLAG antibody.

These five proteins were cloned in a mammalian expression vector with an N-terminal 2Xstrep-TEV-3XFLAG tag for immunoprecipitation with an anti-FLAG antibody, or affinity purification using the streptavidin tag; or tandem purifications for higher purity. The bidirectional plasmid, in addition to the protein of interest, also expresses a GFP protein allowing the easy detection of transfected mammalian cells. A vector encoding the tag alone, and the tagged *E. coli* maltose-binding protein (MBP) were used as a non-RNA binding negative controls; MBP was shown not to bind RNA^49, 50^. We used human Hek-293T cells to achieve transfection efficiencies >90% for all constructs (as determined by flow cytometry, Supplementary Figure S3). We observed various expression efficiencies for these five proteins (Fig. 2A, Supplementary Figure S3), and also observed that expression of PUM2 to some degree may induce cell death. All proteins efficiently purified by anti-FLAG immunoprecipitation, and ENO1 and TIAR were particularly well enriched, based on comparison to input (Fig. 2A, Supplementary Figure S3). These results show that the described expression and purification systems are compatible with a variety of proteins and can be used in a screening workflow.

To capture RNA-protein interactions, we subjected the Hek-293T cells with protein expression constructs to *in vivo* UV-crosslinking. Following immunoprecipitation, the obtained complexes were submitted to a mild treatment with the single-stranded specific RNase I. The RNase treatment is meant to trim larger RNAs leading to more uniform RNA-protein complexes and therefore, a denser signal on the final autoradiograph (Fig. 2B). After separation on a denaturing gel, autoradiography revealed the presence of RNA in association with the purified proteins, as evident from a signal matching the predicted molecular weight of the respective proteins (Fig. 2B). The negative controls, specifically cells transfected with plasmids expressing the tag alone or the tagged MBP, did not exhibit signals on the autoradiograph, confirming the lack of RNA interaction (Fig. 2B). TIAR showed the anticipated CLIP result, in which RNA was recovered only with the crosslinked protein, and the signal formed a smear above the protein’s molecular weight (Fig. 2B)^47^. PUM2 and Larp7 also showed a typical profile, with a crosslink-specific signal (Fig. 2B). Interestingly, for Larp7 we obtained a signal in the non-crosslinked condition, and the signals in both conditions were comparable when applying lower crosslinking energy, suggesting a very stable interaction between Larp7 and RNA (Fig. 2B; and at lower CL energy Supplementary Figure S3). In the conditions used here, we detect a weak signal for the metabolic proteins ENO-1 and SHMT2, despite the efficient purification of ENO1 (Fig. 2B). Taken together, these results show that the CLIP-screening workflow can be applied to a variety of RNA-binding proteins.

### Application of the CLIP-screening workflow to identify putative RNA-binding effectors

Based on the ranking of effectors containing predicted RBDs, thirty-three proteins from four pathogens, specifically *Legionella*, *Salmonella*, *Shigella*, and *Yersinia*, were chosen for a CLIP screen. We selected 18 effectors with relevant RNA-binding function annotations presenting high coverage and similarity scores (Table 2). Additionally, we selected the leucine rich repeats (LRR) containing proteins IpaH, SspH, SlrP, YopM, and LegL1. As described above, these proteins have a tertiary structure similar to TLRs and present conserved lysine and arginine residues that might potentially interact with nucleic acid (Supplementary Figure S2). Albeit lacking a predicted RBD, *Yersinia* YopD has been shown to bind bacterial mRNAs directly^20, 21^, therefore it was selected because it constitutes the only current example of an RNA-binding effector. Finally, *Shigella* IpaC was selected based on its homology to YopD. The selected proteins were cloned in the bidirectional mammalian expression vector and screened for RNA-interaction as described above.

**Figure 3:**
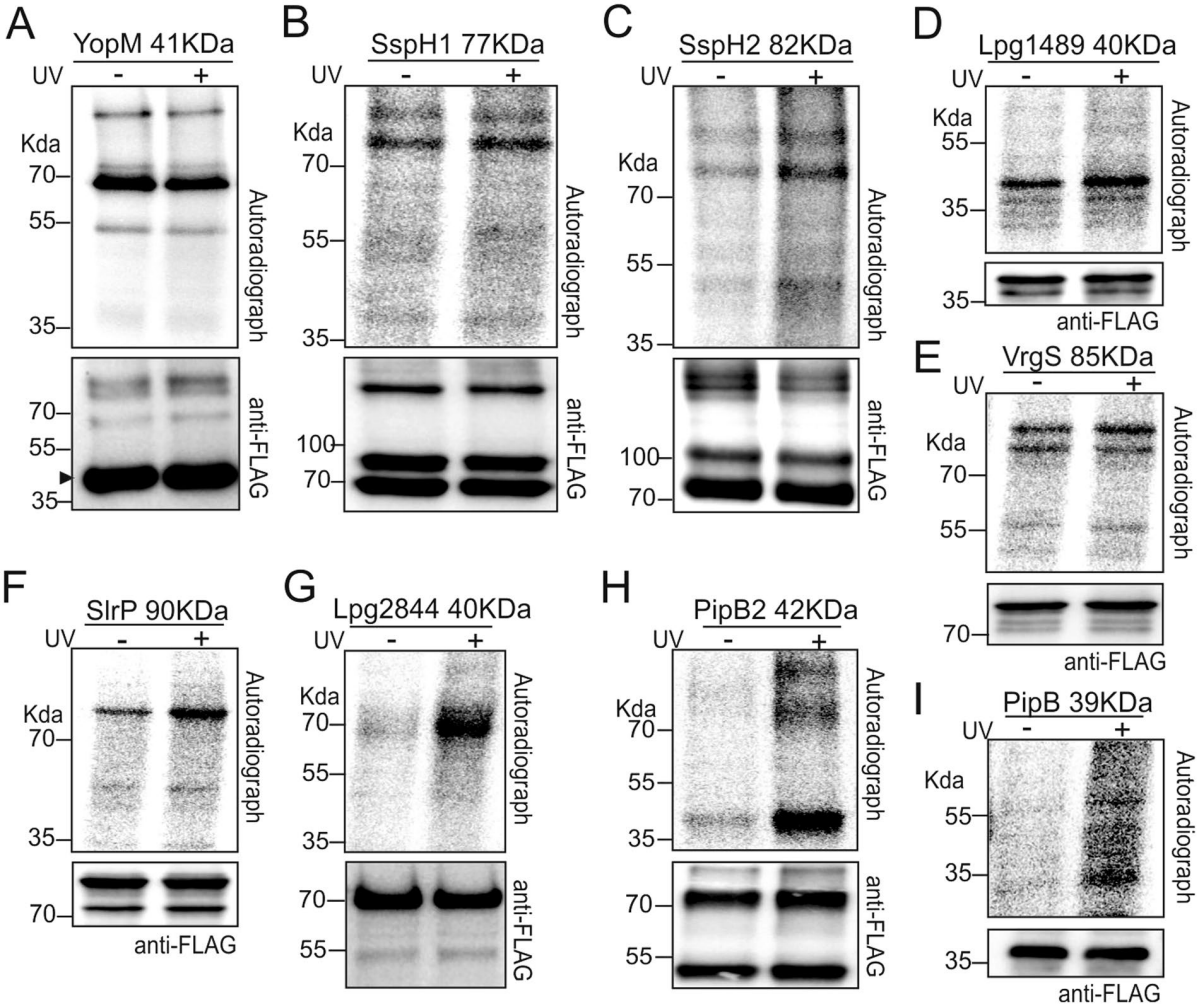
Positive candidates obtained from CLIP-screening. Nine screened effector candidates showed a positive signal on the autoradiograph. (**A–F)** Effectors showing a crosslink signal in the absence and the presence of UV-irradiation. (**G–I)** Effectors showing a crosslink signal only in the presence of UV-irradiation.

Of twenty-eight effectors showing expression in Hek-293 cells, nine effectors, namely YopM, SspH1, SspH2, Lpg1489, VrgS, SlrP, Lpg2844, PipB2, and PipB gave a positive signal on the autoradiograph at the molecular weight corresponding to the respective protein (Fig. 3). Of these, Lpg2844 (Fig. 3G), PipB2 (Fig. 3H) and PipB (Fig. 3I) showed a radioactive signal exclusively after crosslinking, whereas the other six proteins gave signals in both crosslinked and non-crosslinked conditions. Of note, the Western-blot using the anti-FLAG antibody of various immuno-precipitated effector proteins (YopM, SspH1, SspH2, Lpg1489, SlrP, PipB2) showed multiple bands, suggesting the ability of these proteins to form complexes of higher molecular weight despite the denaturing conditions used (Fig. 3). Five effectors (LegC2, LegC8, LepB, Lpg1718, Lpg1290) failed to show expression from the corresponding constructs in Hek-293T cells.

### ATP-interaction and phosphorylation as major sources of CLIP false-positives

**Figure 4:**
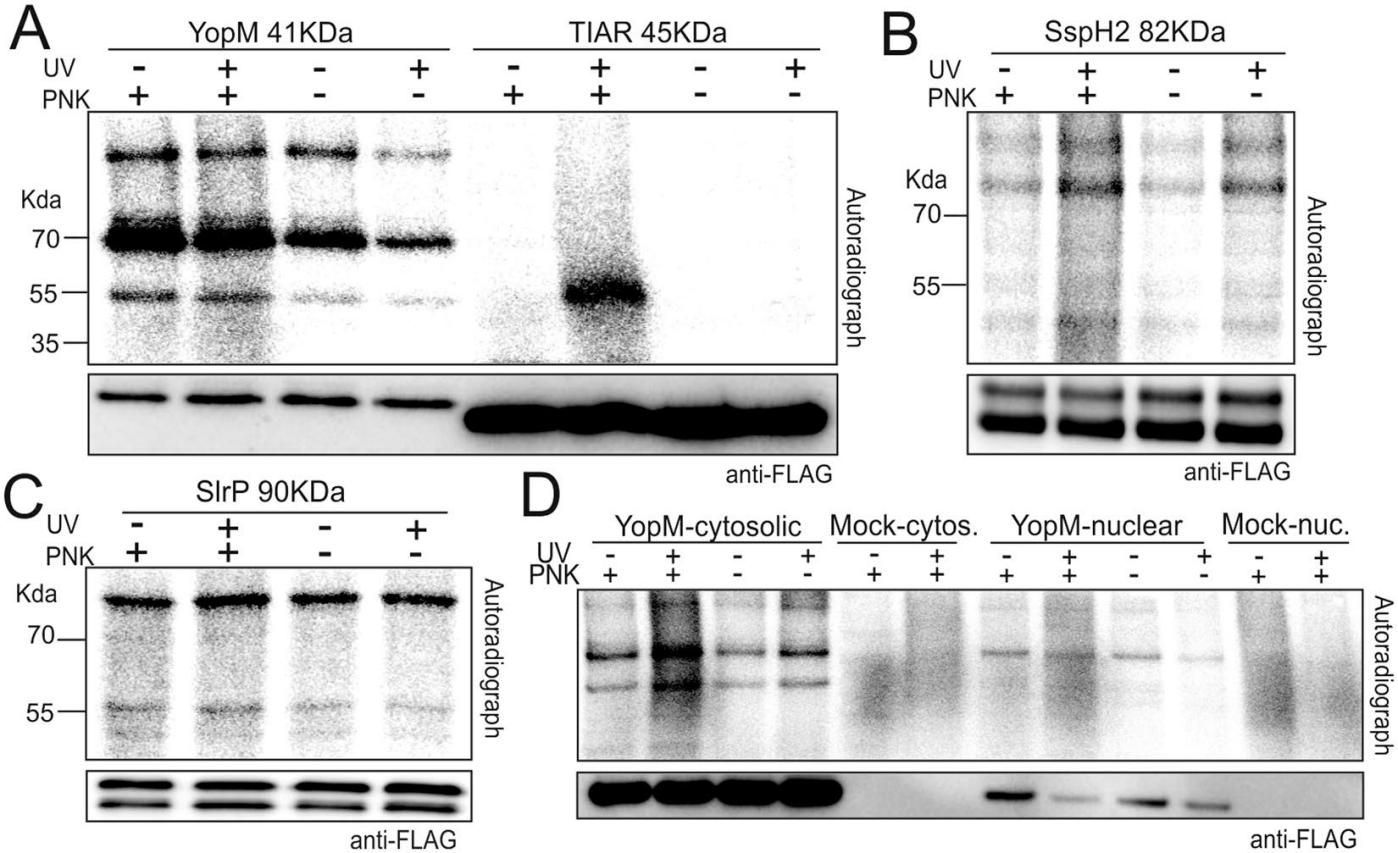
ATP-interaction and phosphorylation as major sources of CLIP false-positives (**A)**. Analysis of YopM autoradiograph signals in the presence and absence of T4 PNK in the labeling reaction. The labeling is independent of PNK activity. TIAR was used as a positive control, the signal with TIAR is lost in the absence of the PNK. (**B**,**C)** Same as in (**A)**. Performed on (**B)**. SspH2. and (**C)**. SlrP. (**D**) YopM was purified from the cytosolic and the nuclear fraction, and analyzed separately for PNK-dependent labeling. Cells expressing the tag alone were used as negative control.

We first explored the group of effectors that gave a radioactive signal in both the UV-crosslinked and non-crosslinked conditions (Fig. 3A–F). On the one hand, a signal without crosslinking may indicate a highly stable protein-RNA interaction, for example, the Larp7-7SK interaction (Fig. 2B, Supplementary Figure S3). On the other hand, the signal obtained in the non-crosslinked conditions may result from a direct interaction with the ATP or a phosphorylation event. To test whether the signal is derived from the bound RNA or results from RNA-independent retention of the γ-phosphate, we investigated YopM, SspH2, and SlrP in more detail (Fig. 3A,C, and F). YopM has been shown to interact with two host kinases, PRK2 and RSK1^51^. Bands of three sizes were detected on the autoradiograph, corresponding to the YopM protein monomer (approx. 53KDa) and the two dimers formed with the kinases (approx. 80KDa and 120KDa with RSK1 and PRK2, respectively) (Fig. 3A). The three bands were detected on the Western blot, with the major band corresponding to the YopM monomer (Fig. 3A, arrowhead). Thus, we hypothesized that kinases co-purifying with YopM may be responsible for the observed signal, labeling the candidate protein by phosphorylation. To test this, we omitted the polynucleotide kinase (PNK) from the labeling reaction, adding only the ATP [Υ-32P]. The three bands were still observed in the absence of the PNK, strongly indicating that they result from the direct phosphorylation of the protein (Fig. 4A). Similarly to YopM, the exclusion of the PNK from the labelling reaction in the SspH2 and SlrP samples did not affect the signal in the autoradiograph, indicating that it is likely derived from phosphorylation of the effectors by host kinases or autophosphorylation (Fig. 4B,C).

Given that YopM localizes in the nucleus of mammalian cells^43, 52^, we evaluated its ability to interact with RNA in this subcellular compartment. The labelling was not dependent on PNK in both nuclear and cytoplasmic fractions (Fig. 4D), arguing that YopM is unable to associate with RNA even in the nucleus. These results show that the use of ATP [Υ-32P] for labelling can misguide the interpretation of CLIP results, for example, for proteins with an ATP-binding capacity and/or if the protein under study forms complexes with ATP-binding proteins. These results highlight the importance of specific controls to address this issue, such as, omitting the PNK from the labelling reaction and considering non-crosslinked controls.

### The behavior of PipB2 and Lpg2844 in CLIP assays suggests a nucleotide-binding activity

Next, we focused on effectors for which a signal was obtained in the crosslinking condition only (PipB, PipB2, and Lpg2844; Fig. 3G–I). In this case, the signal is crosslinking-dependent and unlikely to be caused by direct interaction with the ATP [Υ-32P] or phosphorylation as observed above. Due to a faint signal with PipB, we decided to proceeded further with PipB2 and Lpg2844. Both proteins seemed to form complexes of higher molecular weight, which can correspond to homodimers or an association with host factor(s) (Fig. 3G,H). The crosslink for Lpg2844 was detected only for the higher molecular weight complex (~70 KDa), but for PipB2 the signal predominantly corresponded to the monomer (~42KDa), with weaker signals at higher molecular weight (>70 KDa) (Fig. 3G,H).

**Figure 5:**
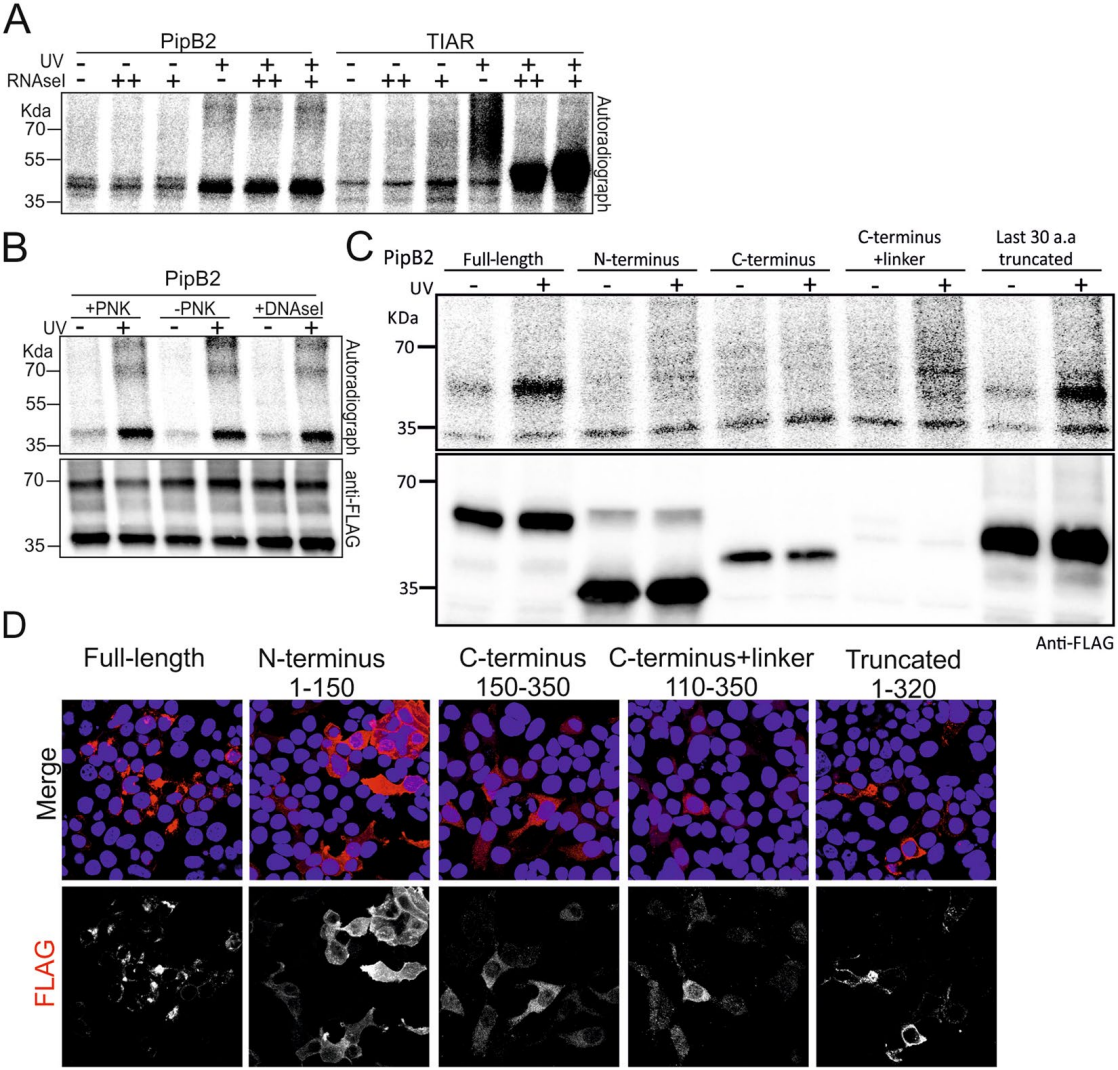
Analysis of effectors showing a UV-dependent crosslink product (**A)**. Using different concentrations of RNase I to test the sensitivity of PipB2 substrates to RNAse digestion. TIAR was used as a positive control. (**B)** DNase I sensitivity assays and PNK-dependent labeling performed on PipB2. (**C**) Analysis of the tagged full-length or truncated domains of PipB2 by CLIP assays. The previously identified pentapeptide motif in the C-terminus is not required for the PipB2 crosslink product. (**B)** Immuno-staining using anti-FLAG (red), showing the sub-cellular localization of PipB2 domains. PipB2 N- and C-terminus are unable to localize correctly. The 1–320 truncated fragment of PipB2 localized comparably to the full-length protein. Cell nuclei are stained with Hoechst (blue).

To investigate the nature of the crosslink obtained with PipB2 and Lpg2844, we first used different concentrations of RNase I to test the sensitivity of the PipB2 and Lpg2844 complexes. There was no difference in the migration patterns for either PipB2 or Lpg2844 complexes in response to different RNase I concentrations (Fig. 5A, Supplementary Figure S4), whereas the TIAR complexes used as control were clearly sensitive to the RNase I treatment (Fig. 5A, Supplementary Figure S4; ref. 47). This result indicates that the complexes formed by PipB2 and Lpg2844 differed from those formed by TIAR that comprise single-stranded RNAs of various length. Nonetheless, it did not fully exclude RNA as a substrate; it is possible that small RNAs, double-stranded RNA, or even DNA are associated with these proteins.

To investigate whether the crosslink results from dsRNA or DNA, we treated the purified PipB2 complexes with benzonase, a nuclease that degrades all forms of RNA and partially DNA, or DNase I a DNA-specific nuclease. Again, no sensitivity to increasing concentrations of these nucleases was observed (Fig. 5B, Supplementary Figure S4). Together these results indicate that the crosslinked and labelled substrates/interacting partner of PipB2 is unlikely to be accessible nucleic acids. Finally, we tested the possibility that the PipB2 and Lpg2844 interacting partner is a small RNA, which would be protected by the proteins and thus not accessible to the enzymes. In this case, the labeling would be dependent on PNK activity. Strikingly, omitting PNK from the labeling reaction did not affect the signal with PipB2 or Lpg2844 suggesting a non-RNA substrate (Fig. 5B, Supplementary Figure S4). To exclude the possibility of a purification linked-artifact, crosslinked and non-crosslinked PipB2 was purified using Strep-tag affinity purification followed by a similar labelling reaction. The results from the affinity purification were the same as with the FLAG-tag, thus excluding a purification-induced artifact (Supplementary Figure S4). The CLIP experiment for PipB2 was also performed in HeLa cells, and a similar result to the Hek-293T cells was obtained (Supplementary Figure S4). Finally, to exclude a UV-induced modification of protein activity or folding, PipB2 was purified from 293 T cells without crosslinking and then the protein was submitted to UV-irradiation prior to labelling, however *in vitro* UV irradiation did not result in a labelled product (Supplementary Figure S4). Overall, our results show that PipB2 and Lpg2844 could have a yet unidentified biochemical function/host factor that is enriched with UV-crosslinking.

To investigate which domain of PipB2 harbors the possible novel biochemical function, PipB2 domains were cloned independently, with the same N-terminal tag, and expressed individually. The 1–150 N-terminal fragment, the 150–350 C-terminal leucine-rich pentapeptide-repeat domain, the 110–350 C-terminus with the upstream linker, and the 1–320 sequence with a truncation of the last 30 amino acids containing the LFNEF functional motif were analyzed similar to the full-length PipB2 (Fig. 5C). Neither the N-terminal nor the C-terminal fragments alone resulted in a crosslink, and the C-terminus with the upstream linker was very weakly expressed (Fig. 5C). Interestingly, the last 30 amino acid truncation did not affect significantly the function leading to a crosslink product (Fig. 5C). Because PipB2 correct localization can affect its function or its interaction with substrates, like the previously identified kinesin-1 linker^42^, we analyzed the localization of the different fragments. The full-length PipB2 accumulated in foci at the periphery of cells as has been shown previously (Fig. 5D; ref. 53). The N and C-terminal fragments showed a diffuse distribution in the cell cytoplasm which argues that both fragments alone loose correct placement in the cells. The 1–320 fragment localized comparably to the full-length protein at membrane foci (Fig. 5D). From these results, we conclude that PipB2 correct localization is necessary to obtain a crosslink-dependent product, and that this function is independent of the previously described pentapeptide motif, arguing a possible novel function for PipB2.

## Discussion

A large number of bacterial effectors have been identified and their characterization has led to important advances in infection and cell biology. Nonetheless, a searchable reference dataset of effectors did not exist when we began this work. We have assembled a gene-name list of all known and predicted effector proteins supported by experimental data. Additionally, our dataset provides information on function, domains, localization, and other features. Our dataset can be a useful resource for other studies on effectors, as well as for training machine-learning approaches for the prediction of secreted effectors in unexplored bacterial genomes^54^. In addition, the information gathered and manually curated here will be useful to complement SecretEPDB, another database for secreted bacterial effectors that was released very recently^55^.

Our bioinformatics analysis of 1,022 unique effector sequences identified putative RBDs with significant scores for 88 proteins. Nine proteins were predicted to contain classical RBDs, such as an RRM similar to the one present in MRN1 was predicted for IpaH9.8, a KH-domain for LepA, and a La RNA-binding domain for Lpg0191. Other predictions involved more ambiguous domains that have been reported to potentially interact with RNA, such as the WD40 domain and the SAM domain^56, 57^. However, these few predicted domains failed to reveal effector-RNA interactions in the subsequent CLIP screen. This could be a limitation of our approach or the cell-line used, or indicate true false-positives among the predicted RBDs. Although “absence of evidence is not evidence of absence”^58^, the prediction of a very small number of RBDs in all known effectors and the lack of RNA detection argues generally for a paucity of RNA targeting by bacterial effectors.

Sequence-based domain assignment requires detectable homology between different stretches of proteins, thus it is possible that effector RBDs have a unique architecture and little similarity to known domains. For example, the TALEs have evolved a unique DNA-binding domain unknown in any other protein^59^. Along the same line, the effector E3 ubiquitine ligases use a domain that is highly distinct from the eukaryotic E3 ligases they mimic functionally (Table 1, refs 2 and 60). Computational approaches taking into consideration the physico-chemical properties of a domain may be more successful in identifying RBDs in effectors; however, such approaches are still not well established. Finally, our computational method could be used for the identification of domains with better conservation patterns, since kinases, phosphatases, and SET domains were successfully identified (Table 1).

In the last decade, CLIP methods have been widely used for the identification of hundreds of protein-RNA interaction sites and unravel the complexity of post-transcriptional regulation. Notwithstanding the success of these approaches, their application should be accompanied by a number of controls that allow the correct interpretation of the results^29, 61^. When applying the CLIP method to screen selected bacterial effector proteins for their ability to interact with RNA, we identified a number of pitfalls. Protein phosphorylation, co-purification of ATP-binding proteins, and molecules susceptible to UV-crosslinking such as free nucleotides, can be erroneously interpreted as interaction with RNA. We found that proteins harboring, or co-purifying with other proteins, having ATP-interacting capacity are labelled during the CLIP procedure. Additionally, proteins interacting with DNA and mono-or dinucleotides can be easily crosslinked to their substrate and subsequently labelled^62, 63^. In our present work, we have developed a simple workflow for the allocation of an RNA-binding activity and the identification of the nature of the substrate. A series of enzymatic approaches such as RNase assays, DNase assays, exclusion of the PNK during labelling, precise molecular weight selection, and negative controls are necessary for the confirmation of an RNA-protein interaction.

We obtained an intriguing result for two effectors, *Salmonella* PipB2 and *Legionella* Lpg2844. These two effectors showed a typical UV-dependent crosslink, but surprisingly the interacting molecule is unlikely to be RNA. This UV-crosslinked molecule(s) can be labeled in the absence of PNK and does not affect significantly the molecular weight of the protein. In addition, we excluded a direct effect of UV-radiation on the protein activity. Rather, we hypothesize that it could be an interaction with a mono- or dinucleotide, considering that free nucleotides can be crosslinked as efficiently as RNA^63^. Considering PipB2 involvement in the regulation of the microtubule network where GTP is an important co-factor for tubulin and other microtubule-regulating proteins, it is possible that PipB2 binds free GTP/GDP^53, 64^. Finally, the discovery of a novel activity for PipB2 independent of the known motifs can serve future studies for the understanding of the manipulation of the microtubule networks by *Salmonella*.

In conclusion, if RNA-binding effectors exist, conservation-based searches may be insufficient to find them. Such effectors might employ novel RBDs, which would evade the current prediction scheme. Future studies to address the fundamental question of whether or not bacteria secreted proteins manipulate host gene expression on the post-transcriptional level may utilize metabolic labeling of host RNAs or RNA baits for the direct capture of interacting effectors.

## Material and Methods

### Bacterial strains, plasmids, and oligonucleotides

Genomic DNA from *Salmonella enterica* serovar Typhimurium SL1344 (strain 14028s was used to clone SspH1), *Shigella flexneri* M90T serotype 5a, *Yersinia pseudotuberculosis*, and *Legionella pneumophila subsp. Pneumophila* Philadelphia-1 was used for the cloning of effector genes. The mammalian expression vector used, pBI-CMV2, was purchased from Clontech. A N-terminal 2X Strep II-TEV-3XFLAG tag was added to pBI-CMV2 by amplifying the tag from pcDNA4/TO-Rev^65^ and inserting it between the BamHI and MluI sites. The oligonucleotides used in this study are described in Table S6. To detect FLAG-tagged proteins the monoclonal ANTI-FLAG® M2 antibody was used (F3165, SIGMA).

### Cell culture, transfection and Flow-Cytometry

Human embryonic kidney cells 293T and Human epithelial HeLa cells (ATCC) were cultured in DMEM GlutaMAX containing 1 g/l glucose (Cat #10567014, Life Technologies), supplemented with 10% fetal bovine serum (cat #S 0115, Biochrom). Cells were maintained at 37 °C in a 5% CO_2_ humidified atmosphere. Cells growing in 10cm dishes were transfected with 10 μg plasmid DNA using Lipofectamine 2000 tranfection reagent (1 μl/1μg DNA; cat# 11668019; Life Technologies), the DNA/Lipofectamine 2000 mix was incubated in reduced serum medium OptiMEM (Cat#31985070; Life Technologies) and added to the cells after 20 min. The transfections were incubated for 48 h. The percentage of transfected cells were analyzed using flow cytometry to detect GFP expression (the Becton-Dickinson Fluorescence activated cell sorter, FACSCalibur). Briefly, cells were detached and collected in 1X PBS + 0.5mM EDTA, washed twice and analyzed.

### CLIP-screen method and Western blotting

After washing with PBS, transfected cells of ~80–90% confluency were placed on ice and irradiated with 0.2 J/cm^2^ UV light at 254–312 nm as previously described^28, 31, 46^. Briefly, cells were harvested by scraping, and lysed in 1X lysis buffer (5X lysis buffer: 50 mM HEPES (pH 7.5), 150 mM KCl, 2 mM EDTA, 1 mM NaF, 1% IGEPAL CA-630) with freshly added 0.5 mM DTT and 1X protease inhibitors cocktail (Sigma, S8820). The lysates were incubated on ice for 20min and centrifuged to pellet debris. The supernatant was incubated with FLAG® M2 Magnetic Beads (Sigma, M8823) or Strep-Tactin Magnetic Beads (Qiagen; 36311) for 3 h at 4 °C with rotation. The supernatant was removed and the beads were washed thoroughly with High-salt buffer (50 mM HEPES (pH 7.5), 500 mM KCl, 0.1% IGEPAL CA-630). RNase I (Ambion) or benzonase (Sigma) was added and incubated at 37 °C for 10min. After cooling on ice for 5min, beads were washed thoroughly with High-salt buffer. For labeling reactions, the beads were washed in PNK buffer (50mM Tris-HCl (pH7.5), 50 mM NaCl, 10 mM MgCl_2_, 5 mM DTT) and subsequently SuperaseIN (Life Technologies, AM2696), PNK, and ATP [Υ-32P] were added to the reaction and incubated at 37 °C for 30 min. Labeling was preceded with a dephosphorylation step using Calf Intestinal Alkaline Phosphatase CIP (NEB, M0290). The beads were then washed in PNK buffer and in High-salt buffer. Crosslinked RNAs and proteins were eluted in gel loading buffer by boiling for 6 min at 90 °C. The eluted complexes were loaded on Bis-Tris 10% polyacrylamide gel and transferred on nitrocellulose membranes, and exposed. The same blots were blocked in 10% milk and incubated with the anti-FLAG antibody to detect the proteins in the corresponding complexes.

For Western blots, aliquots were collected from the lysates before incubation with the beads (input) and from the eluted fraction (eluate). These were loaded on Tris-glycine 10% gel for protein analysis, and transferred on PVDF membranes, the proteins were subsequently detected using the FLAG antibody.

### Sequence alignment, and protein ternary structure analysis

Sequence alignment was performed using Clustal Omega software^66^. For structure analysis, Chimera USCF^67^ and Phyre2^68^ tools were used. Protein structures were collected from the Protein Data Base (PDB).

### Immunofluorescence microscopy

Cells growing on glass coverslips were washed in 1X PBS, fixed with 4% paraformaldehyde (PFA) for 15 min at RT, and permeabilized with 0.5% Triton-X-100 in PBS for 10 min. Blocking was performed for 30 min in 1% Bovine Serum Albumin (BSA) in PBS. Cells were stained with a primary antibody anti-FLAG (1:50, 2 h RT; Sigma) diluted in blocking buffer. Cells were further washed and incubated with the corresponding secondary antibody conjugated with Alexa Flour 594 (1:400, 1 h RT; Life Technologies, A21441). Cell nuclei were stained with Hoechst for 15 min at RT.

### Cellular fractionation

For cytosolic and nuclear fractionation of 293 T cells transfected with plasmids, approx. 4 × 10^7^ cells were collected after crosslinking as described above and cells were pelleted by centrifugation at 1500 g for 3 min at 4 °C. The pellet was resuspended in 1 ml buffer A (10 mM HEPES pH 7.9, 10 mM KCl, 1.5 mM MgCl_2_ 0.34 M sucrose, 10% glycerol, and 1 mM DTT and 1X protease inhibitors cocktail added freshly), SuperaseIn was added, and the suspension was incubated on ice for 5 min. The nuclei were collected by low-speed centrifugation at 1300 g at 4 °C for 4 min, the pellet (nuclear fraction) was washed once in 2ml buffer A, and lysed in buffer B (3 mM EDTA, 0.2 mM EGTA, with 1 mM DTT and protease inhibitors added freshly) on ice for 30 min. The nuclear fraction was cleared from the chromatin by centrifugation at 1700 g for 4min at 4 °C, and the supernatant was saved (nuclear fraction). The supernatant from the low-speed centrifugation was collected (cytosolic fraction), and cleared by high-speed centrifugation at 20 000 g for 15 min at 4 °C. The cytosolic and the nuclear fractions obtained were subsequently used for immuno-precipitation as described above. LaminB and tubulin were detected by western blot and used to normalize the protein concentrations of nuclear and cytoplasmic fractions, respectively.

### APRICOT for RBP-effector prediction

APRICOT was established as an automated pipeline to carry out a sequence-based identification of functional motifs including RNA-binding domains in the bacterial effectors compiled from various studies. APRICOT uses two main data sources, namely Conserved Domain Database (CDD) and InterPro that consist of conserved motifs, signatures and functional domains from various databases. CDD comprise of 50,648 entries (February 2016) as Position Specific Scoring Matrix (PSSM) and InterPro comprise of 28926 entries (February 2016) as Hidden Markov Models (HMM) or position weight matrix. As shown in the Table S4, based on the RBDs known from the eukaryotic studies, the pipeline compiles a set reference RNA-binding domains (RBDs) from the aforementioned databases. The bacterial effectors are subjected to the analysis by RPS-BLAST and InterProScan tools, which search for conserved motifs in their corresponding sequences from CDD and InterPro database respectively. The domain prediction statistics, which include parameters like domain coverage, similarity, identity and E-value, are evaluated for the selection of candidate RBPs among the queries. Thereafter, those effectors are selected as candidate RBPs that are predicted with at least one of the reference RBDs and share considerable sequence conservation (domain coverage > 30% and similarity >25%) with their corresponding reference domains^32^. These candidate RBPs are further annotated by sequence-based features like chemical properties, protein compositions and structural properties, which are compared with the references in order to calculate similarity scores for each of these features. These similarity scores are further used for the ranking of selected candidates in order to differentiate proteins that consist of RBDs with high biological conservations than the proteins that are predicted with RBDs of lower conservations. The source-code and related documentations for APRICOT are freely available online at https://github.com/malvikasharan/APRICOT.

## Electronic supplementary material

Combined Supplementary material

Supplementary Table S2

Supplementary Table S3

Supplementary Table S4

Supplementary Table S5

Supplementary Table S7
