# Supplementary material for: A systematic analysis of the RNA-targeting potential of secreted bacterial effector proteins
Source: Sci Rep. 2017 Aug 24;7:9328. doi: 10.1038/s41598-017-09527-0 (PMC5570926; doi:10.1038/s41598-017-09527-0)
Supplement: Supplementary file 1 — Combined Supplementary material [file 41598_2017_9527_MOESM1_ESM.pdf]

A systematic analysis of the RNA-targeting potential of secreted bacterial effector proteins

Caroline Tawk[1], Malvika Sharan[1#], Ana Eulalio, Jörg Vogel[1,2*]

SUPPLEMENTARY FIGURES

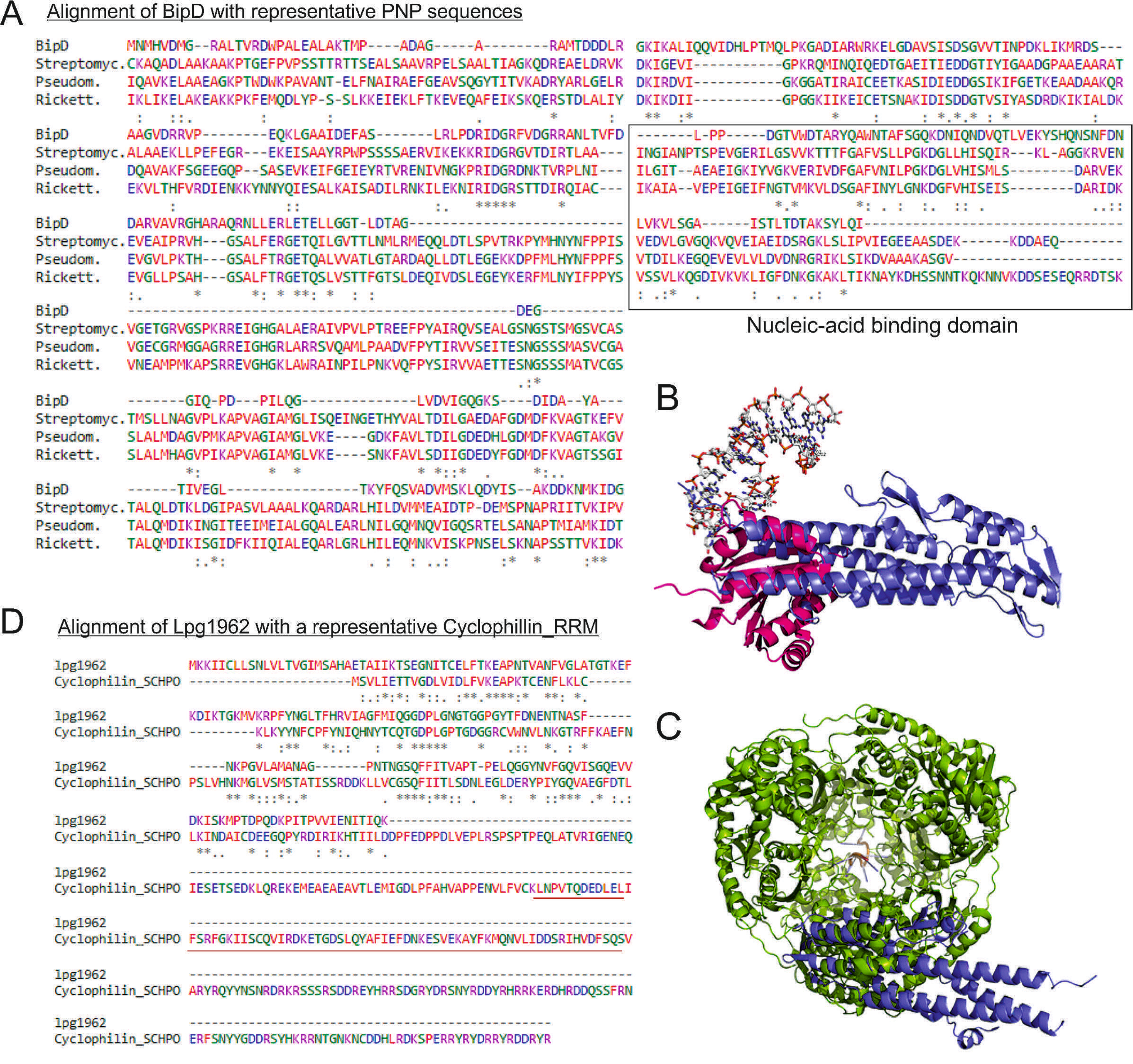
A
Alignment of BipD with representative PNP sequences
BipD MNMHVDMG--RALTVRDWPALEALAKTMP----ADAG-----A--------RAMTDDDLR
Streptomyc. CKAQADLAAKAAKPTGEFPVPSSTTRTTSEALSAAVRPELSAALTIAGKQDREAELDRVK
Pseudom. IQAVKELAAEAGKPTWDWKPAVANT-ELFNAIRAEFGEAVSQGYTITVKADRYARLGELR
Rickett. IKLIKELAKEAKKPKFEMQDLYP-S-SLKKEIEKLFTKEVEQAFEIKSKQERSTDLALIY
BipD AAGVDRRVP--------EQKLGAAIDEFAS-------LRLPDRIDGRFVDGRRANLTVFD
Streptomyc. ALAAEKLLPEFEGR---EKEISAAYRPWPSSSSAERVIKEKKRIDGRGVTDIRTLAA---
Pseudom. DQAVAKFSGEEGQP--SASEVKEIFGEIEYRTVRENIVNGKPRIDGRDNKTVRPLNI---
Rickett. EKVLTHFVRDIENKKYNNYQIESALKAISADILRNKILEKNIRIDGRSTTDIRQIAC---
BipD DARVAVRGHARAQRNLLERLETELLGGT-LDTAG---------------------------
Streptomyc. EVEAIPRVH---GSALFERGETQILGVTTLNMLRMEQQLDTLSPVTRKPYMHNYNFPPIS
Pseudom. EVGVLPKTH---GSALFTRGETQALVVATLGTARDAQLLDTLEGEKKDPFMLHYNFPPFS
Rickett. EVGLLPSAH---GSALFTRGETQSLVSTTFGTSLDEQIVDSLEGEYKERFMLNYIFPPYS
BipD ---DEG---------
Streptomyc. VGETGRVGSPKRREIGHGALAERAIVPVLPTREEFPYAIRQVSEALGSNGSTSMGSVCAS
Pseudom. VGECGRMGGAGRREIGHGRLARRSVQAMLPAADVFPYTIRVVSEITESNGSSSMASVCGA
Rickett. VNEAMPMKAPSRREVGHGKLAWRAINPILPNKVQFPYSIRVVAETTESNGSSSMATVCGS
BipD -------GIQ-PD---PILQG-------------LVDVIGQGKS----DIDA--YA---
Streptomyc. TMSLLNAGVPLKAPVAGIAMGLISQEINGETHYVALTDILGAEDAFGDMDFKVAGTKEFV
Pseudom. SLALMDAGVPMKAPVAGIAMGLVKE----GDKFAVLTDILGDEDHLGDMDFKVAGTAKGV
Rickett. SLALMHAGVPIKAPVAGIAMGLVKE----SNKFAVLSDIIGDEDYFGDMDFKVAGTSSGI
BipD ------TIVEGL---------------TKYFQSVADVMSKLQDYIS--AKDDKNMKIDG
Streptomyc. TALQLDTKLDGIPASVLAAALKQARDARLHILDVMMEAIDTP-DEMSPNAPRIITVKIPV
Pseudom. TALQMDIKINGITEEIMEIALGQALEARLNILGQMNQVIGQSRTELSANAPTMIAMKIDT
Rickett. TALQMDIKISGIDFKIIQIALEQARLGRLHILEQMNKVISKPNSELSKNAPSSTTVKIDK
BipD GKIKALIQQVIDHLPTMQLPKGADIARWRKELGDAVSISDSGVVTINPDKLIKMRDS---
Streptomyc. DKIGEVI-----------GPKRQMINQIQEDTGAEITIEDDGTIYIGAADGPAEAARAT
Pseudom. DKIRDVI-----------GKGGATIRAICEETKASIDIEDDGSIKIFGETKEAADAAKQR
Rickett. DKIKDII-----------GPGGKIIKEICETSNAKIDISDDGTVSIYASDRDKIKIALDK
BipD -------L-PP-----DGTVWDTARYQAWNTAFSGQKDNIQNDVQTLVEKYSHQNSNFDN
Streptomyc. INGIANPTSPEVGERILGSVVKTTTFGAFVSLLPGKDGLLHISQIR---KL-AGGKRVEN
Pseudom. ILGIT--AEAEIGKIYVGKVERIVDFGAFVNILPGKDGLVHISMLS--------DARVEK
Rickett. IKAIA--VEPEIGEIFNGTVMKVLDSGAFINYLGNKDGFVHISEIS--------DARIDK
BipD LVKVLSGA------ISTLTDTAKSYLQI---------------------------------
Streptomyc. VEDVLGVGQKVQVEIAEIDSRGKLSLIPVIEGEEAASDEK------KDDAEQ---------
Pseudom. VTDILKEGQEVEVLVLDVDNRGRIKLSIKDVAAAKASGV--------------------
Rickett. VSSVLKQGDIVKVKLIGFDNKGKAKLTIKNAYKDHSSNNTKQKNNVKDDSESEQRRDTSK
Nucleic-acid binding domain
B
C
D
Alignment of Lpg1962 with a representative Cyclophillin_RRM
lpg1962 MKKIICLLSNLVLTVGIMSAHAETAIIKTSEGNITCELFTKEAPNTVANFVGLATGTKEF
Cyclophilin_SCHPO ---------------------MSVLIETTVGDLVIDLFVKEAPKTCENFLKLC------
lpg1962 KDIKTGKMVKRPFYNGLTFHRVIAGFMIQGGDPLGNGTGGPGYTFDNENTNASF------
Cyclophilin_SCHPO ---------KLKYYNFCPFYNIQHNYTCQTGDPLGPTGDGGRCVWNVLNKGTRFFKAEFN
lpg1962 -----NKPGVLAMANAG-------PNTNGSQFFITVAPT-PELQGGYNVFGQVISGQEVV
Cyclophilin_SCHPO PSLVHNKMGLVSMSTATISSRDDKLLVCGSQFIITLSDNLEGLDERYPIYGQVAEGFDTL
lpg1962 DKISKMPTDPQDKPITPVVIENITIQK---------------------------------
Cyclophilin_SCHPO LKINDAICDEEGQPYRDIRIKHTIILDDPFEDPPDLVEPLRSPSPTPEQLATVRIGENEQ
lpg1962 --
Cyclophilin_SCHPO IESETSEDKLQREKEMEAEAEAVTLEMIGDLPFAHVAPPENVLFVCKLNPVTQDEDLELI
lpg1962 --
Cyclophilin_SCHPO FSRFGKIISCQVIRDKETGDSLQYAFIEFDNKESVEKAYFKMQNVLIDDSRIHVDFSQSV
lpg1962 --
Cyclophilin_SCHPO ARYRQYYNSNRDRKRSSSRSDDREYHRRSDGRYDRSNYRDDYRHRRKERDHRDDQSSFRN
lpg1962 ---
Cyclophilin_SCHPO ERFSNYYGDDRSYHKRRNTGNKNCDDHLRDKSPERRYRYDRRYRDDRYR

Figure S1. Alignment and structural analysis excludes false-positive candidates

A. Alignment of protein sequences of three representative PNPase proteins and the BipD *Burkholderia* effector. Colors indicate residues with similar physicochemical properties. '*' identical residues, ':' indicates residues with similar properties, and '.' indicates identical residues among a subset of proteins. BipD lacks homology with the nucleic-binding domain of PNPases in the C-terminus (boxed). **B.** Superimposition of BipD in blue (PDB 2J9T; [1]) with PNPase in pink bound to RNA in CPK (PDB 4AM3; [2]). **C.** Superimposition of BipD in blue with PNPase in green bound to RNA in CPK (PDB 3GCM; [3]). **D.** Alignment of the protein sequences of Lpg1962 and an RRM-containing cyclophilin from *Saccharomyces*. Lpg1962 lacks the domain homologous to the cyclophilin-RRM (underlined in red).

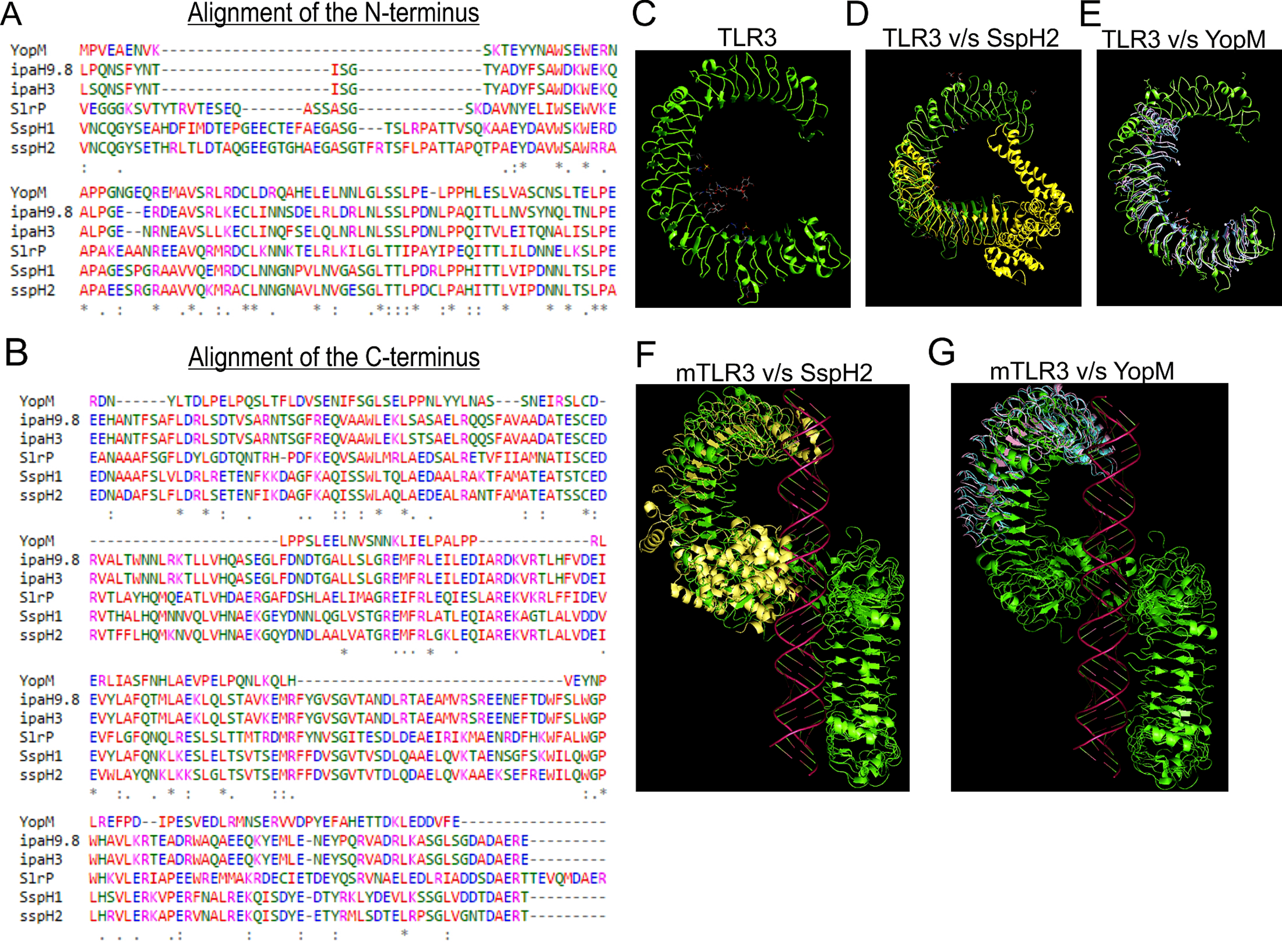

A
Alignment of the N-terminus
YopM MPVEAENVK-----------------------------------SKTEYYNAWSEWERN
ipaH9.8 LPQNSFYNT-------------------ISG-------------TYADYFSAWDKWEKQ
ipaH3 LSQNSFYNT-------------------ISG-------------TYADYFSAWDKWEKQ
SlrP VEGGGKSVTYTRVTESEQ-------ASSASG------------SKDAVNYELIWSEWVKE
SspH1 VNCQGYSEAHDFIMDTEPGEECTEFAEGASG---TSLRPATTVSQKAAEYDAVWSKWERD
sspH2 VNCQGYSETHRLTLDTAQGEEGTGHAEGASGTFRTSFLPATTAPQTPAEYDAVWSAWRRA
YopM APPGNGEQREMAVSRLRDCLDRQAHELELNNLGLSSLPE-LPPHLESLVASCNSLTELPE
ipaH9.8 ALPGE--ERDEAVSRLKECLINNSDELRLDRLNLSSLPDNLPAQITLLNVSYNQLTNLPE
ipaH3 ALPGE--NRNEAVSLLKECLINQFSELQLNRLNLSSLPDNLPPQITVLEITQNALISLPE
SlrP APAKEAANREEAVQRMRDCLKNNKTELRLKILGLTTIPAYIPEQITTLILDNNELKSLPE
SspH1 APAGESPGRAAVVQEMRDCLNNGNPVLNVGASGLTTLPDRLPPHITTLVIPDNNLTSLPE
sspH2 APAEESRGRAAVVQKMRACLNNGNAVLNVGESGLTTLPDCLPAHITTLVIPDNNLTSLPA
B
Alignment of the C-terminus
YopM RDN------YLTDLPELPQSLTFLDVSENIFSGLSELPPNLYYLNAS---SNEIRSLCD-
ipaH9.8 EEHANTFSAFLDRLSDTVSARNTSGFREQVAAWLEKLSASAELRQQSFAVAADATESCED
ipaH3 EEHANTFSAFLDRLSDTVSARNTSGFREQVAAWLEKLSTSAELRQQSFAVAADATESCED
SlrP EANAAAFSGFLDYLGDTQNTRH-PDFKEQVSAWLMRLAEDSALRETVFIIAMNATISCED
SspH1 EDNAAAFSLVLDRLRETENFKKDAGFKAQISSWLTQLAEDAALRAKTFAMATEATSTCED
sspH2 EDNADAFSLFLDRLSETENFIKDAGFKAQISSWLAQLAEDEALRANTFAMATEATSSCED
YopM ---------------------LPPSLEELNVSNNKLIELPALPP-------------RL
ipaH9.8 RVALTWNNLRKTLLVHQASEGLFDNDTGALLSLGREMFRLEILEDIARDKVRTLHFVDEI
ipaH3 RVALTWNNLRKTLLVHQASEGLFDNDTGALLSLGREMFRLEILEDIARDKVRTLHFVDEI
SlrP RVTLAYHQMQEATLVHDAERGAFDSHLAELIMAGREIFRLEQIESLAREKVKRLFFIDEV
SspH1 RVTHALHQMNNVQLVHNAEKGEYDNNLQGLVSTGREMFRLATLEQIAREKAGTLALVDDV
sspH2 RVTFFLHQMKNVQLVHNAEKGQYDNDLAALVATGREMFRLGKLEQIAREKVRTLALVDEI
YopM ERLIASFNHLAEVPELPQNLKQLH-----------------------------VEYNP
ipaH9.8 EVYLAFQTMLAEKLQLSTAVKEMRFYGVSGVTANDLRTAEAMVRSREENEFTDWFSLWGP
ipaH3 EVYLAFQTMLAEKLQLSTAVKEMRFYGVSGVTANDLRTAEAMVRSREENEFTDWFSLWGP
SlrP EVFLGFQNQLRESLSLTTMTRDMRFYNVSGITESDLDEAEIRIKMAENRDFHKWFALWGP
SspH1 EVYLAFQNKLKESLELTSVTSEMRFFDVSGVTVSDLQAAELQVKTAENSGFSKWILQWGP
sspH2 EVWLAYQNKLKKSLGLTSVTSEMRFFDVSGVTVTDLQDAELQVKAAEKSEFREWILQWGP
YopM LREFPD--IPESVEDLRMNSERVVDPYEFAHETTDKLEDDVFE-----------------
ipaH9.8 WHAVLKRTEADRWAQAEEQKYEMLE-NEYPQRVADRLKASGLSGDADAERE---------
ipaH3 WHAVLKRTEADRWAQAEEQKYEMLE-NEYSQRVADRLKASGLSGDADAERE---------
SlrP WHKVLERIAPEEWREMMAKRDECIETDEYQSRVNAELEDLRIADDSDAERTTEVQMDAER
SspH1 LHSVLERKVPERFNALREKQISDYE-DTYRKLYDEVLKSSGLVDDTDAERT---------
sspH2 LHRVLERKAPERVNALREKQISDYE-ETYRMLSDTELRPSGLVGNTDAERT---------
C
TLR3
D
TLR3 v/s SspH2
E
TLR3 v/s YopM
F
mTLR3 v/s SspH2
G
mTLR3 v/s YopM

Figure S2. Structural analysis predicting RNA-binding effector proteins

A. Alignment of the N-terminal protein sequences of six E3 ubiquitine ligase effectors. Colors indicate residues with similar physicochemical properties. '*' indicates identical residues, ':' indicates residues with similar properties, and '.' indicates identical residues among a subset of proteins. **B.** Alignment of the C-terminal sequence of the same proteins as in **A**. Symbols and colors are the same as in **A**. **C.** Structure of the Toll-like receptor 3 (TLR3) ligand-binding domain PDB accession number 2A0Z[4]. The backbone is rendered as cartoon and residues important for binding are rendered as sticks. **D.** Superimposition of SspH2 in yellow (PDB 3G06; [5]) with TLR3 in green (PDB 1ZIW; [6]) (RMSD 2.269). **E.** Superimposition of YopM in blue and violet (PDB 1JL5, 1G9U;[7]) with TLR3 in green (RMSD 2.244, 2.015). **F.** Superimposition of YopM same as in **E** with mouse TLR3 dimer in green bound to double-stranded RNA in pink (PDB 3CIY;[8]) (RMSD 2.342, 2.142). **G.** Superimposition of SspH2 same as in **D** with mouse TLR3 dimer bound to double-stranded RNA (RMSD 2.293). Images and structure superimposition were performed using CHIMERA-UCSF.

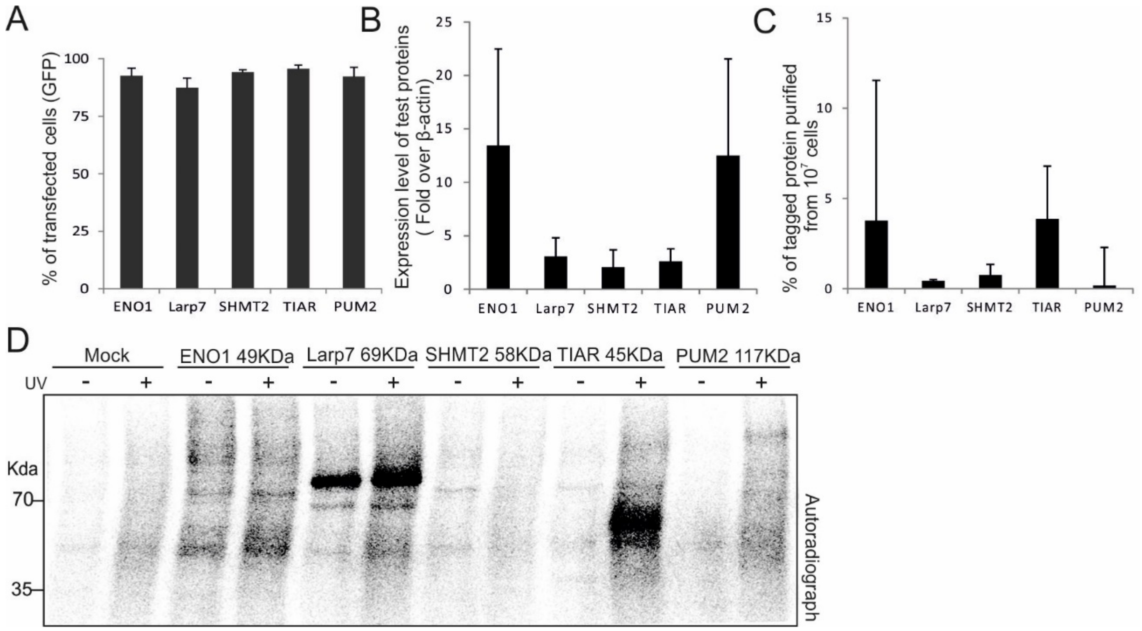

Figure S3. Development of the CLIP-screening workflow using an RBP test-set

A. Transfected GFP-positive HEK293T cells measured by flow-cytometry. Transfections were incubated for 48h. **B.** The relative abundance of the expressed proteins compared to β-actin. **C.** The relative proportion of purified protein compared to the input. All experiments are representative of at least three biological replicates. Error bars represent standard deviations (SD). **D.** Autoradiograph of streptavidin purified Strep-FLAG tagged test-set proteins, showing Larp7-RNA association in the absence and the presence of UV-crosslinking (UV-irradiation at 280-315 nm).

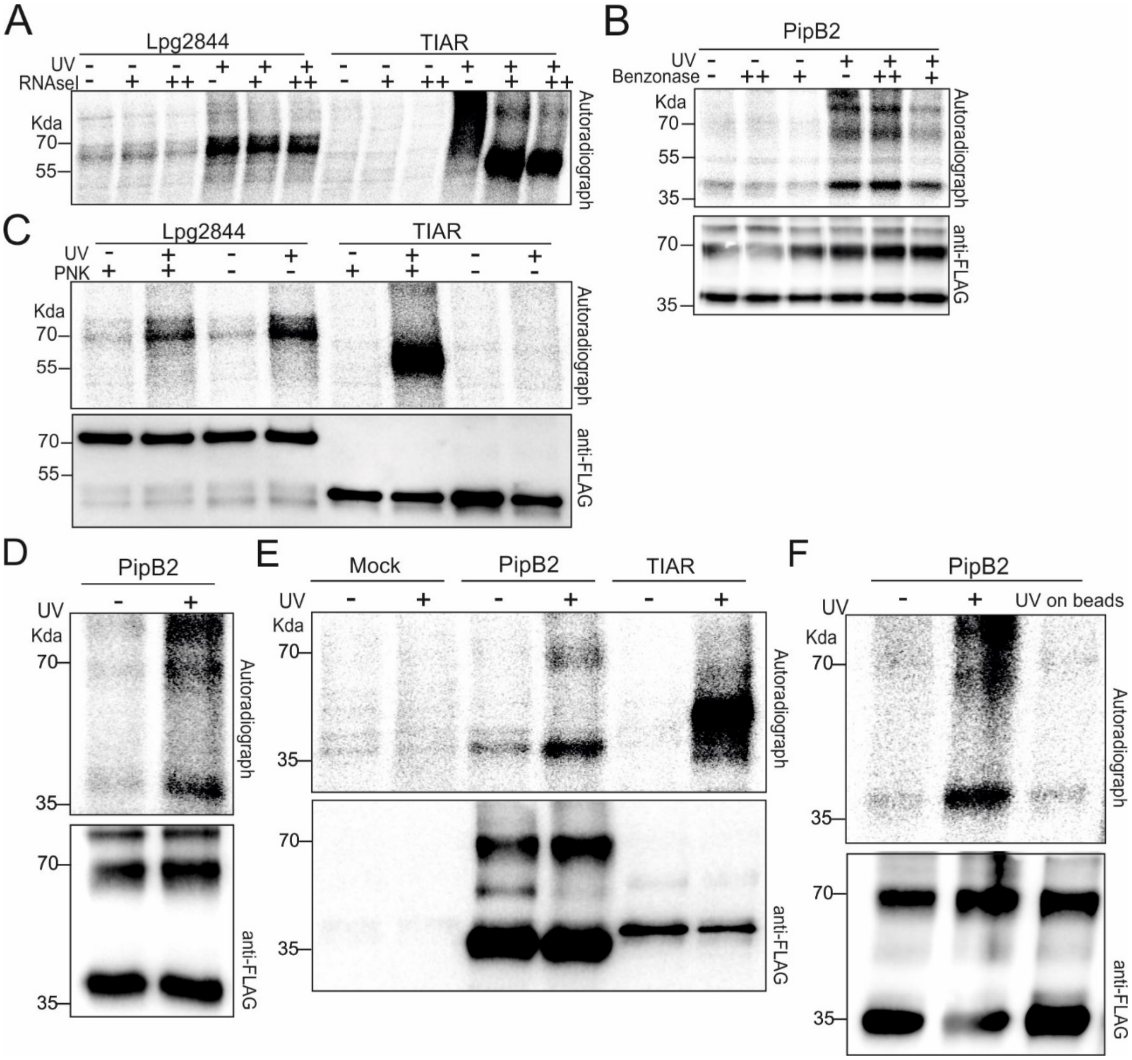

Figure S4. Analysis of effectors showing a UV-dependent crosslink product

A. Using different concentrations of RNase I to test the sensitivity of Lpg2844 substrates to RNAse digestion. TIAR was used as a positive control. **B.** Benzonase sensitivity assays performed on PipB2. **C.** PNK-dependent labeling test performed on Lpg2844. TIAR was used as positive control. **D.** PipB2 purified using the Strep-tag and analyzed by CLIP assays. The results obtained are the same as with FLAG-tag purification. **E.** CLIP assays with PipB2 in HeLa cells using the FLAG-tag purification, shows same results as in HEK293T cells. TIAR was used as a positive control, and the tag alone as a negative control. **F.** PipB2 was purified

using the FLAG-tag from non-irradiated cells and then submitted to UV *in vitro* prior to labelling. Standard CLIP analysis of PipB2 was used as control (–UV and +UV).

SUPPLEMENTARY REFERENCES

1 Johnson, S. *et al.* Self-chaperoning of the type III secretion system needle tip proteins IpaD and BipD. *The Journal of biological chemistry* **282**, 4035-4044, doi:10.1074/jbc.M607945200 (2007).

2 Hardwick, S. W., Gubbey, T., Hug, I., Jenal, U. & Luisi, B. F. Crystal structure of Caulobacter crescentus polynucleotide phosphorylase reveals a mechanism of RNA substrate channelling and RNA degradosome assembly. *Open biology* **2**, 120028, doi:10.1098/rsob.120028 (2012).

3 Nurmohamed, S., Vaidialingam, B., Callaghan, A. J. & Luisi, B. F. Crystal structure of Escherichia coli polynucleotide phosphorylase core bound to RNase E, RNA and manganese: implications for catalytic mechanism and RNA degradosome assembly. *Journal of molecular biology* **389**, 17-33, doi:10.1016/j.jmb.2009.03.051 (2009).

4 Bell, J. K. *et al.* The molecular structure of the Toll-like receptor 3 ligand-binding domain. *Proceedings of the National Academy of Sciences of the United States of America* **102**, 10976-10980, doi:10.1073/pnas.0505077102 (2005).

5 Quezada, C. M., Hicks, S. W., Galan, J. E. & Stebbins, C. E. A family of Salmonella virulence factors functions as a distinct class of autoregulated E3 ubiquitin ligases. *Proceedings of the National Academy of Sciences of the United States of America* **106**, 4864-4869, doi:10.1073/pnas.0811058106 (2009).

6 Choe, J., Kelker, M. S. & Wilson, I. A. Crystal structure of human toll-like receptor 3 (TLR3) ectodomain. *Science* **309**, 581-585, doi:10.1126/science.1115253 (2005).

7 Evdokimov, A. G., Anderson, D. E., Routzahn, K. M. & Waugh, D. S. Unusual molecular architecture of the Yersinia pestis cytotoxin YopM: a leucine-rich repeat protein with the shortest repeating unit. *Journal of molecular biology* **312**, 807-821, doi:10.1006/jmbi.2001.4973 (2001).

8 Liu, L. *et al.* Structural basis of toll-like receptor 3 signaling with double-stranded RNA. *Science* **320**, 379-381, doi:10.1126/science.1155406 (2008).

9 Wang, Z. *et al.* iCLIP predicts the dual splicing effects of TIA-RNA interactions. *PLoS biology* **8**, e1000530, doi:10.1371/journal.pbio.1000530 (2010).

10 Hafner, M. *et al.* Transcriptome-wide identification of RNA-binding protein and microRNA target sites by PAR-CLIP. *Cell* **141**, 129-141, doi:10.1016/j.cell.2010.03.009 (2010).

11 Markert, A. *et al.* The La-related protein LARP7 is a component of the 7SK ribonucleoprotein and affects transcription of cellular and viral polymerase II genes. *EMBO reports* **9**, 569-575, doi:10.1038/embor.2008.72 (2008).

12 Castello, A. *et al.* Insights into RNA biology from an atlas of mammalian mRNA-binding proteins. *Cell* **149**, 1393-1406, doi:10.1016/j.cell.2012.04.031 (2012).

SUPPLEMENTARY TABLES

Table S1

35 bacterial pathogen/symbiont genera	
Acidovorax	*Herbaspirillum*
Aeromonas	*Legionella*
Agrobacterium	*Mesorhizobium*
Anaplasma	*Orientia*
Bartonella	*Pantoea*
Bordetella	*Photorhabdus*
Bradyrhizobium	*Pseudomonas aeruginosa*
Brucella	*Pseudomonas syringae*
Burkholderia	*Ralstonia*
Campylobacter	*Rhizobium*
Chlamydia	*Rickettsia*
Citrobacter	*Salmonella*
Coxiella	*Shigella*
Ehrlichia	*Sinorhizobium*
Erwinia	*Vibrio*
Escherichia	*Xanthomonas*
Francisella	*Yersinia*
Helicobacter	

Table S6. Test-set of human RNA-binding proteins

Human protein	Molecular weight with tag	Function	RNA targets	Study
TIAR	45 KDa	mRNA chaperone, involved in splicing	67,002 introns, 2,277 ncRNAs and 8,602 3'UTRs	[9]
Pumilio 2	116 KDa	Sequence-specific RNA-binding protein that regulates translation and mRNA stability	~3,000 unique transcripts	[10]
Larp7	72 KDa	Binds to 7SK RNA to form transcription regulation RNP complex	Known to bind 7SK RNA and form 7SK ribonucleoprotein	[11]
Enolase 1	50 KDa	Metabolic enzyme involved in glycolysis and gene regulation	Shown to bind around 115 transcripts	[12]
SHMT2	50 KDa	Metabolic enzyme involved in glycine synthesis	Shown to bind around 350 transcripts	[12]
